# Short-Term Low Temperature Induces Nitro-Oxidative Stress that Deregulates the NADP-Malic Enzyme Function by Tyrosine Nitration in *Arabidopsis thaliana*

**DOI:** 10.3390/antiox8100448

**Published:** 2019-10-01

**Authors:** Juan C. Begara-Morales, Beatriz Sánchez-Calvo, María V. Gómez-Rodríguez, Mounira Chaki, Raquel Valderrama, Capilla Mata-Pérez, Javier López-Jaramillo, Francisco J. Corpas, Juan B. Barroso

**Affiliations:** 1 Group of Biochemistry and Cell Signaling in Nitric Oxide, Department of Experimental Biology, Center for Advanced Studies in Olive Grove and Olive Oils, Faculty of Experimental Sciences, University of Jaén, Campus “Las Lagunillas”, s/n, E-23071 Jaén, Spain; jbegara@ujaen.es (J.C.B.-M.); bscalvo@ujaen.es (B.S.-C.); mvgomez@ujaen.es (M.V.G.-R.); mounira@ujaen.es (M.C.); ravalde@ujaen.es (R.V.); mmata@ujaen.es (C.M.-P.); 2 Institute of Biotechnology, Department of Organic Chemistry, Faculty of Sciences, University of Granada, E-18071 Granada, Spain; fjljara@ugr.es; 3 Group of Antioxidants, Free Radicals, and Nitric Oxide in Biotechnology, Food and Agriculture, Department of Biochemistry, Cell and Molecular Biology of Plants, Estación Experimental del Zaidín, CSIC, C/Profesor Albareda 1, E-18080 Granada, Spain; javier.corpas@eez.csic.es

**Keywords:** NADP malic enzyme, low temperature, nitric oxide, tyrosine nitration, peroxynitrite, reactive oxygen species, reactive nitrogen species, nitro-oxidative stress

## Abstract

Low temperature (LT) negatively affects plant growth and development via the alteration of the metabolism of reactive oxygen and nitrogen species (ROS and RNS). Among RNS, tyrosine nitration, the addition of an NO_2_ group to a tyrosine residue, can modulate reduced nicotinamide-dinucleotide phosphate (NADPH)-generating systems and, therefore, can alter the levels of NADPH, a key cofactor in cellular redox homeostasis. NADPH also acts as an indispensable electron donor within a wide range of enzymatic reactions, biosynthetic pathways, and detoxification processes, which could affect plant viability. To extend our knowledge about the regulation of this key cofactor by this nitric oxide (NO)-related post-translational modification, we analyzed the effect of tyrosine nitration on another NADPH-generating enzyme, the NADP-malic enzyme (NADP-ME), under LT stress. In *Arabidopsis thaliana* seedlings exposed to short-term LT (4 °C for 48 h), a 50% growth reduction accompanied by an increase in the content of superoxide, nitric oxide, and peroxynitrite, in addition to diminished cytosolic NADP-ME activity, were found. In vitro assays confirmed that peroxynitrite inhibits cytosolic NADP-ME2 activity due to tyrosine nitration. The mass spectrometric analysis of nitrated NADP-ME2 enabled us to determine that Tyr-73 was exclusively nitrated to 3-nitrotyrosine by peroxynitrite. The in silico analysis of the *Arabidopsis* NADP-ME2 protein sequence suggests that Tyr73 nitration could disrupt the interactions between the specific amino acids responsible for protein structure stability. In conclusion, the present data show that short-term LT stress affects the metabolism of ROS and RNS, which appears to negatively modulate the activity of cytosolic NADP-ME through the tyrosine nitration process.

## 1. Introduction

Low temperature (LT) is an environmental issue that affects plant physiology and biochemistry at different levels, including antioxidant enzymes, photosynthesis, gene expression, nutrients, and water uptake [1,2,3,4,5,6,7,8,9]. At the economic level, the impact of LT on plant crops, such as pepper, rice, tomato, maize, and olive, and tropical and subtropical fruits worldwide, is very significant as it influences both their production and quality [1,10,11]. However, model plants, such as *Arabidopsis thaliana,* act as a very useful tool to decipher the molecular mechanism of response to LT stress [12,13,14,15,16].

LT usually induces nitro-oxidative stress, mediated by the overproduction of reactive oxygen(ROS) and nitrogen (RNS) species [1]. Interestingly, an increasing number of reports suggest that certain reduced nicotinamide-dinucleotide phosphate(NADPH)-generating dehydrogenases might be involved in the protection mechanism against nitro-oxidative stresses induced by adverse environmental conditions [17,18,19,20,21,22,23]. In plants, several NADPH-generating systems come into play, such as ferredoxin-NADP reductase as a component of photosystem I, and a group of NADP-dehydrogenases that have been found in different subcellular locations. This group of enzymes includes NADP-isocitrate dehydrogenase (NADP-ICDH), glucose-6-phosphate dehydrogenase (G6PDH), 6-phosphogluconate dehydrogenase (6PGDH) and the NADP-malic enzyme (NADP-ME), also called NADP-malate dehydrogenase.

The NADP-malic enzyme, together with the other NADP-dehydrogenases, is a key component of the NADPH-production systems required to maintain the redox balance in cells. It has been identified from bacteria to humans as an enzyme that catalyzes the reversible oxidative decarboxylation of l-malate to pyruvate, CO_2_, and NADPH [24,25,26]. In plants, different isoenzymes have been described in plastids and cytosol. In *Arabidopsis,* cytosolic NADP-ME2 is considered to be responsible for most NADP-ME activity in mature tissues [27,28,29] and has been linked to a wide range of processes [30], such as lignin biosynthesis, by providing NADPH [31], and to control cytosolic pH by balancing the synthesis and degradation of l-malate [32]. Other roles that have been suggested for NADP-ME include the control of stomatal closure through the degradation of l-malate during the daytime and seed germination [33]. The presence of a cytosolic NADP-ME isoform has been reported in the guard cell complexes of C_3_ plant wheat. However, a more profound analysis of the NADP-ME isoforms in plants is still required. These studies will contribute to unraveling the biological role of plastidic and cytosolic isoenzymes in the same tissue, or even different NADP-MEs in the same subcellular location. Four NADP-ME isoforms have been identified in monocot rice (*Oryza sativa*) [34] that, unfortunately, have not yet been characterized at the molecular level. In addition, the transcripts of different NADP-ME isoforms, located in the cytosol and photosynthetic and non-photosynthetic organelles, have been identified in *Flaveria* sp., which exhibit different C_3_ and C_4_ photosynthetic pathways [35]. Interestingly, NADP-ME has also been proposed to be involved in plant responses to biotic and abiotic stress (reviewed by [30]).

One of the regulatory mechanisms of plant response to stress is protein function modulation via nitric oxide (NO)-related posttranslational modifications (PTMs) [36,37,38]. Interestingly, different NADPH-generating enzymes have been identified as being the target of these NO-PTMs [39,40,41], but information on the specific impact of these modifications to their function in the nitro-oxidative stress context is still scarce. Along this line, LT is one of the main abiotic stresses that modulates the metabolism of ROS and RNS, and also affects NADP-ME function [1], which suggests the regulation of this enzyme by NO-PTMs, such as tyrosine nitration, as reported for NADPH-generating systems [41,42]. S-nitrosylation, the attachment of NO to a specific cysteine residue, is an NO-PTMs that has been widely analyzed as a regulatory process during plant response to stress [43]. However, tyrosine nitration also appears to play an important role during plant response to the nitro-oxidative stress generated under environmental insults [44]. This NO-PTM is produced by the addition of a nitro group (-NO_2_) to the tyrosine residue aromatic ring which gives rise to 3-nitrotyrosine. This results in significantly reducing local pKa, which can affect the tyrosine function [45]. Different factors have been proposed to regulate this PTM, including protein structure and environmental compartments. Although information on specific denitrase activity in plants that allow this PTM to be considered key in signaling processes is still lacking [46], these covalent changes may result in effects such as protein function loss and gain or no functional change [42,47,48,49,50] and, therefore, impact cellular function. Indeed, different NADPH-generating enzymes have been proposed to be modulated by tyrosine nitration [41,42], but the effect of NO on protein structure [42] has been analyzed only for NADP-ICDH, with NADP-ME2 being one of the least studied enzymes.

In this context, the main goals of this study were to determine whether LT stress could affect the cross-talk between ROS and RNS metabolisms in *Arabidopsis thaliana* seedlings and to study its potential correlation with the group of NADPH-generating dehydrogenases. As NADP-ME was the only NADP-dehydrogenase to be modulated under LT conditions, we focused on this enzyme to study the potential regulatory effect of tyrosine nitration by an in vitro approach.

## 2. Materials and Methods

### 2.1. Plant Material and Growth Conditions

*Arabidopsis thaliana* ecotype Columbia seeds were surface-sterilized and grown on Petri dishes containing Murashige and Skoog medium (Sigma-Aldrich-Fluka, St. Louis, MO, USA) according to [51]. *Arabidopsis* seeds were subsequently grown for 2 weeks in a 16 h light/dark regime at 22 °C/8 h and 18 °C (long-day conditions) with a light intensity of 50 µE m^−2^ s^−1^. For cold treatment purposes, the Petri dishes containing *Arabidopsis* seedlings were placed inside different containers with ice and transferred to a cold room set at 4 ± 2 °C for 48 h.

### 2.2. Crude Extracts of Plant Tissues

*Arabidopsis thaliana* seedlings were frozen and ground in liquid N_2_ using a pestle and mortar. Then an extraction buffer (1:3, *w/v*) containing 100 mM Tris-HCl, pH 7.8, 0.02 (*v/v*) Triton X-100,1mM EDTA and 10% (*v/v*) glycerol was added. The resulting homogenates were centrifuged at 17,000× *g* for 20 min, and supernatants were subjected to different analyses.

### 2.3. Detection of Nitric Oxide (NO), Peroxynitrite (ONOO^−^) and Superoxide Radicals (O_2_^•−^) by Confocal Laser Scanning Microscopy 

The root tips of *Arabidopsis thaliana* were used to detect different reactive oxygen and nitrogen species by confocal laser scanning Microscopy (CLSM,) according to [52]. Briefly, samples were incubated in the dark for 1h with the following specific fluorescents freshly made in 10 mM Tris-HCl, pH 7.4: 10 µM 4-amino-5-methylamino-2´,7´-difluoro fluorescein diacetate (DAF-FM DA, Calbiochem, San Diego, CA, USA) to detect nitric oxide(NO); 10 µM 3-(*p*-aminophenyl) fluorescein (APF, Invitrogen, Carlsbad, CA, USA) to detect peroxynitrite (ONOO^−^) and 10 µM dihydroethidium (DHE, Sigma-Aldrich-Fluka, St. Louis, MO, USA) to detect superoxide radicals (O_2_^•−^). DAF-FM DA and APF were incubated at 25 °C and DHE at 37 °C. Then samples were washed twice in the same buffer for 15 min and mounted on a microscope slide for examination purposes with a CLSM (Leica TCS SP5 II). The following filters and collection modalities were used: 490 nm excitation and 520 nm emission for DHE, 495 nm excitation and 515 nm emission for DAF-FM DA, and 495 nm excitation and 515 nm emission for APF. The images obtained by CLSM from the control and LT-treated *Arabidopsis* roots were processed and analyzed by the Leica confocal software [52].

### 2.4. Enzyme Activity Assay

Glucose-6-phosphate dehydrogenase (G6PDH; EC 1.1.1.49), NADP-isocitrate dehydrogenase (NADP-ICDH, EC 1.1.1.42) and NADP-malic enzyme (NADP-ME; EC 1.1.1.40) activity were determined at 25 °C according to [17,23,53] by spectrophoto metrically recording the reduction in NADP at 340 nm. The reaction mixture (1 mL) contained 50 mM 4-(2-Hydroxyethyl)piperazine-1-ethanesulfonic acid (HEPES), pH 7.6, 2 mM MgCl_2_, and 0.8 mM NADP. The reaction was initiated by adding 5 mM glucose-6-phosphate, 10 mM 2R,3S-isocitrate or 1 mM l-malate, respectively.

### 2.5. Expression and Purification of Cytosolic Arabidopsis Thaliana NADP-Dependent Malic Enzyme 2 (NADP-ME2)

c-DNA was obtained from the total *Arabidopsis* leaf RNA by using the first strand c-DNA synthesis kit (Roche, Basel, Switzerland) following the manufacturer’s instruction. Then the amplification of the cytosolic NADP-ME2 gene (NM_121205.3) was performed by PCR using Fast Start High-Fidelity polymerase (Roche, Basel, Switzerland) and specific primer sets: 5′-AGAGATATGGGAAGTACTCCGACTGATTTACC-3′ and 5′-ACAAAACTTTTTTAACGGTAGTTTCTGTACACAG-3′. The PCR product (1785 bp) was then cloned into the pALEXb vector (BiomedalSL, Sevilla, Spain) and the expression of the recombinant protein carrying an *N*-terminal choline-binding domain was performed according to [48]. Briefly, the recombinant protein was induced by adding 1 mM salicylate and 10 mM 3-methyl benzoate. Protein purification was then carried out using a 1-mL LYTRAP column (Biomedal SL, Sevilla, Spain), in which protein was eluted in 1-mL fractions using a discontinuous gradient of choline [48]. Finally, the purity grade of the recombinant protein expression was analyzed by 10% SDS-PAGE.

### 2.6. Treatment of NADP-ME with SIN-1 (Peroxynitrite Donor)

Molecule SIN-1 (3-morpholinosyl-nonimine) is considered a peroxynitrite donor that is a protein-nitrating compound [54]. Cytosolic recombinant NADP-ME2 was incubated at 37 °C for 1 h with increasing concentrations (0 to 5 mM) of SIN-1 (Calbiochem, San Diego, CA, USA) freshly made before use. A polyclonal antibody against 3-nitrotyrosine (dilution 1:2500) was employed to corroborate the nitration of NADP-ME2 by the immunoblot analysis.

### 2.7. Identification of Nitrated Tyrosine in Recombinant Cytosolic NADP-ME2 by Mass Spectrometric Techniques 

To identify the potential targets of tyrosine nitration, the purified recombinant cytosolic NADP-ME2 treated with SIN-1 was processed by MS/MS as previously described [42]. The sample was subjected to a reduction with dithiothreitol (DTT), derivatization with iodoacetamide (IAA), and enzymatic digestion with trypsin (37 °C, 8 h). It was subsequently purified to eliminate the interferences from the digestion process. Then the peptide mixture was analyzed in a MALDI-TOF/TOF (matrix-assisted laser desorption ionization-time of flight/time of flight) mass spectrometer (4800, AB Sciex) to evaluate sample quality. To this end, peptide mass fingerprinting (PMF) and Uniprot databases were used to interpret the MALDI-TOF spectra and for identification purposes (release 2011_02), respectively. The resulting sample was subjected to a liquid chromatography–tandem mass spectrometry (LC-MS/MS) analysis, using a Velos-LTQ mass spectrometer equipped with a micro-ESI ion source (Thermo Fisher, San Jose, CA, USA). Briefly, the sample was evaporated and subsequently diluted in water containing methanol (5%) and acid formic (1%). Next a 10-cm long, 150-μm id Vydac C18 column (Vydac, IL, USA) was used for the chromatographic process, in which separation was performed at 1 μL min^−1^ with a 3% to 40% acetonitrile gradient for 30 min (solvent A, 0.1% formic acid; solvent B, acetonitrile with 0.1% formic acid). The Velos-LTQ instrument was operated in the positive ion mode with a 2 kV spray voltage. The scan range of each full MS scan was *m/z* 400 to 2000. After each MS scan, the targeted MS/MS spectra were collected to identify both the unmodified and nitrated form of the predicted tyrosine-containing peptides. The parent mass list of the targeted scan was selected to ensure maximum coverage of the tyrosine-containing tryptic peptides for ME. To obtain a potential list of targeted *m/z* values, the protein was subjected to in silico digestion using nitrated tyrosine as a dynamic modification. The resulting list of predicted peptides (in both the nitrated and unmodified forms) was filtered to exclude any peptide not containing tyrosine residues. The MS/MS spectra were searched with the Proteome Discoverer software (Thermo Fisher, San Jose, CA, USA) under the following conditions: peptide mass tolerance 2 Da, fragment tolerance 0.8 Da, enzyme set as trypsin, no missed cleavages. The dynamic modifications were cysteine carbamidomethylation (+57 Da), methionine oxidation (+16 Da), and tyrosine nitration (+45). Searches were carried out using a database containing all the peptides listed in Table S1. Identifications were filtered with XCorr > 3, P(pep) < 15%. The MS spectra of the nitrated tyrosines were manually validated by comparing the spectra obtained for the unmodified peptide and the nitrated peptide.

### 2.8. Modeling and the Molecular Evolution Analysis

The tertiary structure of the NADP-malic enzyme from *Arabidopsis thaliana* was modeled at the Swiss Model Server [55], the CPHmodels 2.1 server [56]. HHpred [57], IntFold [58], I-Tasser [59], Phyre2 [60], and RaptorX [61]. Model quality was evaluated by three-dimensional profiles (Verify 3D) [62], the distribution of atom–atom contacts (Errat method) [63], Procheck [64], Qmean [65], and Qmean-Z score [66]. The coordinates of the quaternary structure were calculated by super positioning the best model on the Protein Data Bank (PDB)entry 1O0S with XtalView [67].

The Evolutionary Trace Server was employed to analyze molecular evolution [68] using the model of the tertiary structure as input. Both phylogenetic significance and evolutionary conservation were explored by a BLASTP [69] search the UniProtKB [70] release 2014_10. The prediction of pKa was carried out by PropKa 3.0 [71], and the phosphorylation score was computed on the NetPhos 2.0 Server [72].

### 2.9. Other Assays

Protein concentration was estimated by the Bio-Rad Protein Assay (Hercules, CA, USA) using bovine serum albumin as a standard. The statistical significance between means was analyzed by a Student’s *t*-test.

## 3. Results

**Figure 1:**
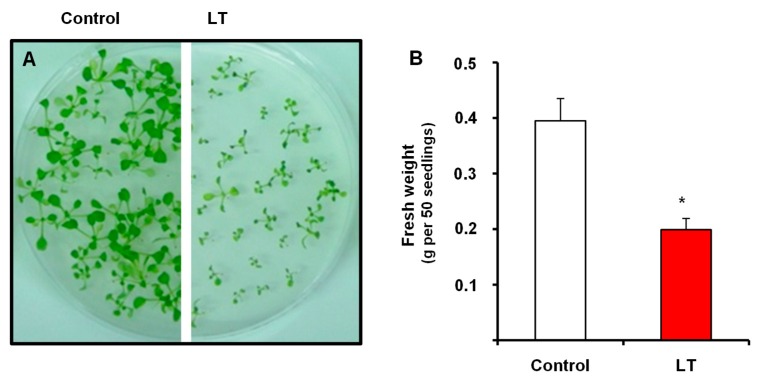
(**A**) Appearance and (**B**) fresh weight of the 14-day-old *Arabidopsis* seedlings grown in MS medium subjected to low temperature (LT) for the last 48 h. The results are the mean of at least three different experiments + SEM. For each experiment, 200 seedlings were used. * Differences in relation to the control values were significant at *p* < 0.05.

Figure 1A shows the phenotype of the 14-day-old *Arabidopsis thaliana* seedlings grown in MS medium subjected to low temperature (LT) for the last 48 h. These seedlings were smaller in size, and the leaves presented some chlorotic symptoms. The fresh weight of these seedlings also drastically reduced by 50% as a result of LT (Figure 1B), which indicates that the LT conditions caused significant stress to *Arabidopsis thaliana* seedlings.

### 3.1. NADP-Dehydrogenase Activities under Short-Term LT Stress

**Figure 2:**
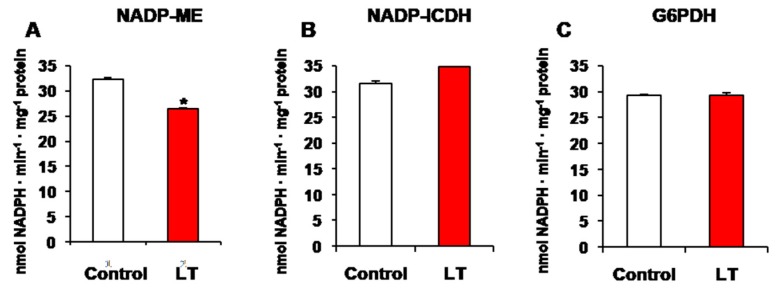
Activity of NADP-dehydrogenase enzymes of the 14-day-old *Arabidopsis* seedlings grown in MS medium subjected to low temperature (LT) for the last 48 h. (**A**) NADP-malic enzyme (NADP-ME). (**B**) NADP-isocitrate dehydrogenase (NADP-ICDH). (**C**) Glucose-6-phosphate dehydrogenase. The results are the mean of at least three different experiments + SEM. * Differences in relation to the control values were significant at *p* < 0.05. NADP: nicotinamide-dinucleotide phosphate.

It has been previously reported that, as part of the recycling system of NADPH, several NADP-dehydrogenases may be implicated in plant response to some abiotic conditions [23,30,73]. Hence, the activity of the main NADP-dehydrogenases at LT was evaluated. Figure 2A shows how NADP-ME lowered by 25% at LT, whereas a slight, but insignificant increase was recorded for NADP-ICDH (Figure 2B). G6PDH activity showed no change at LT (Figure 2C).

### 3.2. Cellular Analysis of Superoxide Radical (O_2_^•−^), Nitric Oxide (NO), and Peroxynitrite (ONOO^−^) in the Roots of the Arabidopsis Seedlings Exposed to LT

**Figure 3:**
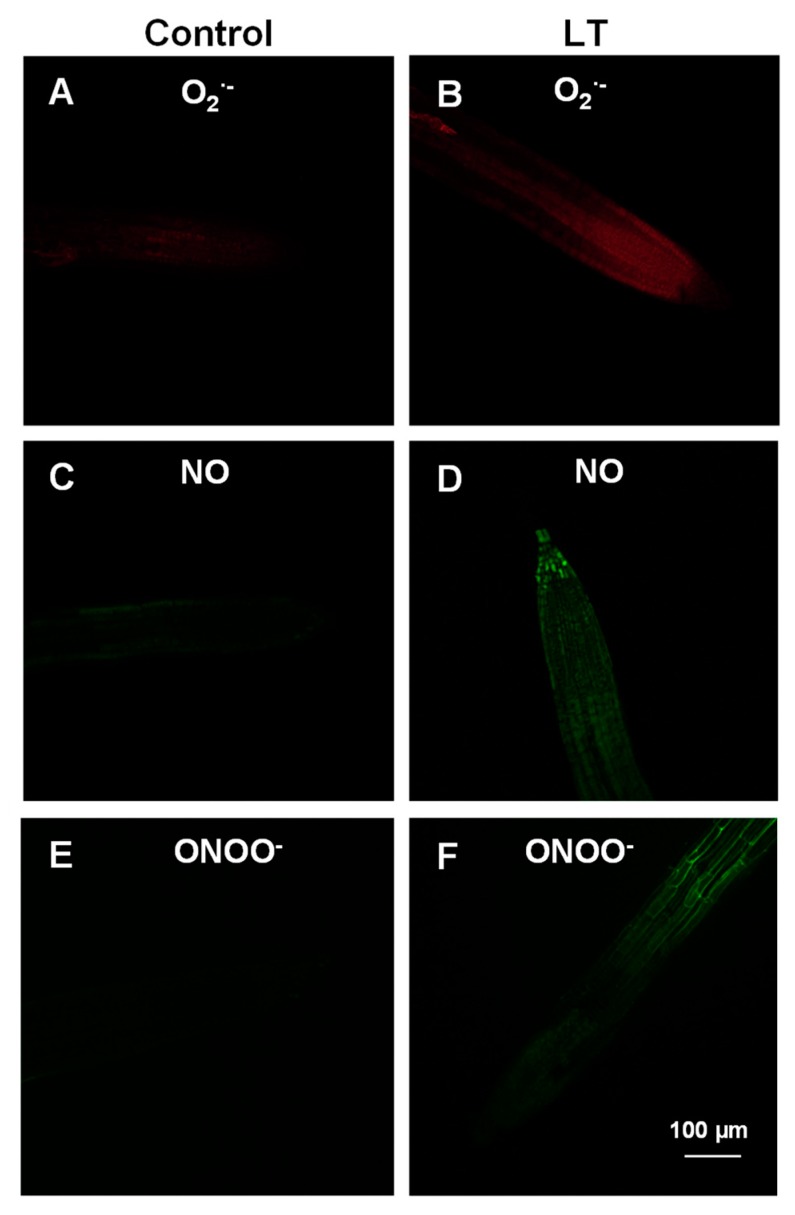
Fluorescence and confocal images showing the in vivo detection of superoxide radical (O_2_^•−^) (panels **A** and **B**), nitric oxide (NO) (panels **C** and **D**), and peroxynitrite (ONOO^−^) (panels **E** and **F**) in *Arabidopsis* root tips after exposing seedlings to low temperature (LT) for 48 h.

Figure 3 (panels A–F) illustrates the examination by CLSM of the levels of O_2_^•−^, NO, and ONOO^−^ in the root tips of *Arabidopsis thaliana* seedlings after LT stress for 48 h. O_2_^•−^ content was evaluated after incubating *Arabidopsis* seedlings with fluorescent probe DHE. In the roots of the control seedlings, slight green fluorescence related to O_2_^•−^ was observed in the tips (Figure 3A). However, increased green fluorescence was detected after exposing seedlings to LT (Figure 3B). Regarding NO production, a significant increase was detected in the roots of the seedlings exposed to LT by using DAF-FM as the fluorescence probe compared to the control plants (Figure 3C,D). APF was used to analyze ONOO^−^ content, which resulted from the reaction between O_2_^•−^ and NO. In the control roots, ONOO^−^ showed a very slight fluorescent signal (Figure 3E). Nevertheless, this RNS notably increased as a result of LT stress, with homogeneous distribution throughout roots (Figure 3F), similarly to the observed NO distribution.

### 3.3. Expression and Purification of Cytosolic NADP-ME2. Effect of Peroxynitrite (ONOO^−^)

**Figure 4:**
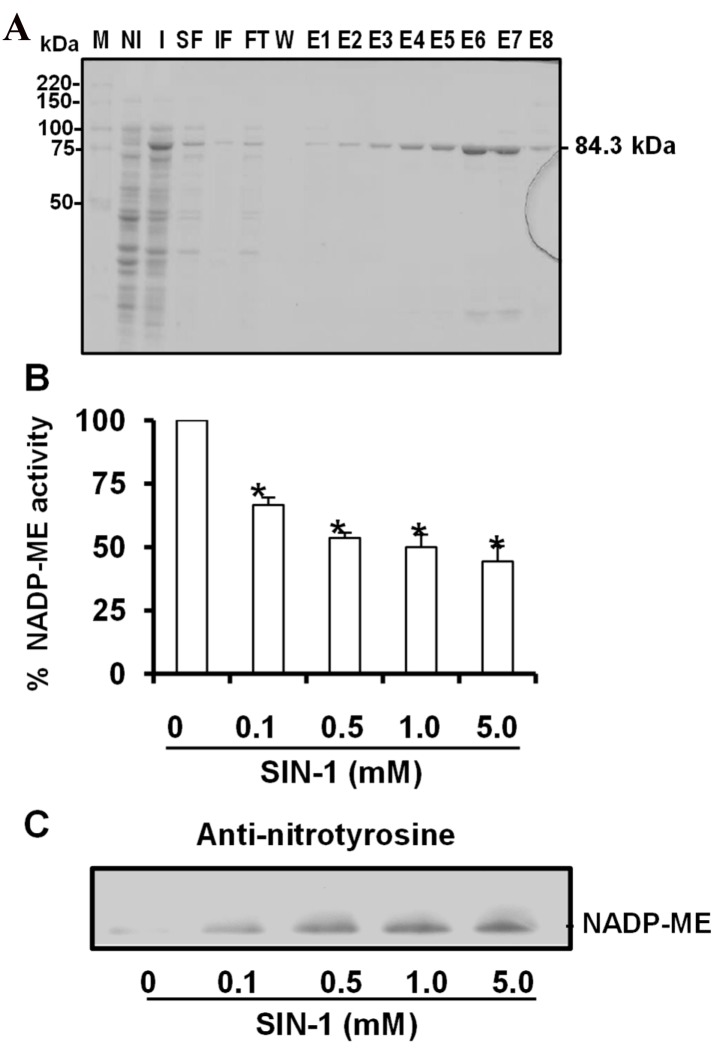
Purification of recombinant cytosolic NADP-ME2 and effect of SIN-1 (3-morpholinosydnonimine (peroxynitrite donor)) on its activity. (**A**) Purity of recombinant NADP-ME after expression and purification was evaluated by SDS-PAGE (10%) and Coomasie blue staining. M, molecular markers; NI, total protein in no-induced culture; I, total protein in induced culture; SF, soluble fraction; IF, insoluble fraction; FT, flow-through; W, wash; E1-E8, elution fractions. (**B**) Effect of SIN-1 on recombinant NADP-EM2 activity. (**C**) Representative immunoblot showing the degree of tyrosine nitration of NADP-EM2 treated with different concentrations of SIN-1 and detected with an antibody against 3-nitrotyrosine (dilution 1:2500). 3 µg of protein was used per line. The specific activity of recombinant NADP-ME was 9850 µmol NADPH min^−1^ mg^−1^ protein. Data are the means ± SEM of at least three replicates. * Differences from the control values were significant at *p* < 0.05.

As NADP-ME was the only NADP-dehydrogenase to be modulated under LT stress, and to gain further insight into cytosolic *Arabidopsis thaliana* NADP-ME2, which is the most abundant in this plant [74], the recombinant protein was obtained by cloning the cDNA encoding the enzyme and overexpression in *Escherichia coli*. Figure 4A shows the different fractions obtained after the expression and affinity purification of LYTAG recombinant NADP-ME. The molecular weight of this recombinant construction was 84.3 kDa and resulted from the cytosolic NADP-ME2 protein (63 kDa) containing Ly-tag (21.28 kDa). The fractions with an acceptable purity grade (E5 to E7) showed NADP-EM activity of 9850 µmol NADPH min^−1^ mg^−1^ protein and were used for the subsequent experiments. To analyze the potential action of peroxynitrite generated under LT stress, the recombinant protein was treated with SIN-1 (3-morpholinosydnonimine) as the peroxynitrite donor. Figure 4B depicts the inhibitory effect of ONOO^−^ on NADP-ME activity in a dose-dependent manner, which ranged from 33% with 0.1 mM SIN-1 to 56% with 5 mM SIN-1. An immunoblot analysis, run with an antibody against 3-nitrotyrosine, was employed to corroborate the nitration of recombinant NADP-ME as a result of SIN-1 treatment (Figure 4C).

### 3.4. Spectral Characterization of the Nitrated Recombinant Arabidopsis NADP-ME2

**Figure 5:**
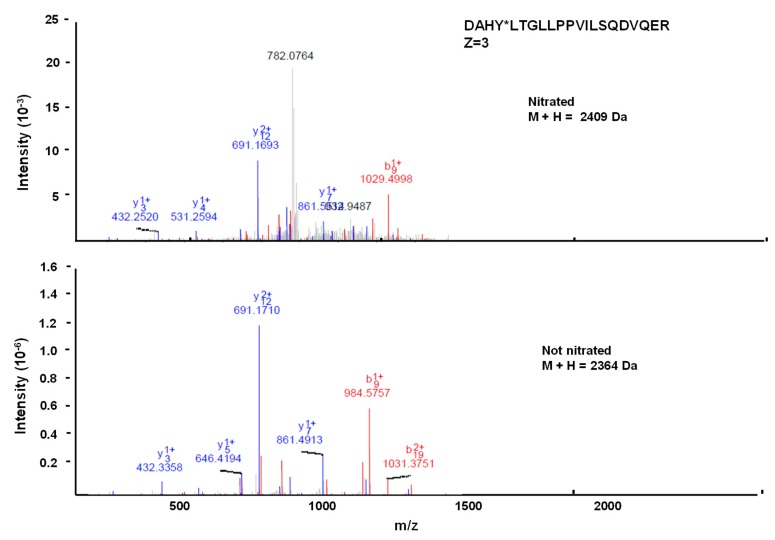
Comparison of the nitrated (top) and unmodified (bottom) MS/MS spectra of the identified peptide (DAHY*LTGLLPPVILSQDVQER) from *Arabidopsis thaliana* NADP-ME2. Peptide fragment ions are indicated by “b” if the charge is retained on the *N*-terminus (red) and by “y” if the charge is maintained on the *C*-terminus (blue). The subscript indicates the number of amino acid residues in the considered fragment from either *N*-terminus or *C*-terminus. The superscript indicates the charge (1+ or 2+) to the backbone fragmentation.

There are 25 tyrosine residues in the sequence of the cytosolic NADP-ME2 in *Arabidopsis* plants. Thus to be able to identify which of these residues is(are) (a) target(s) of this NO-related post-translational modification, the recombinant protein was treated with peroxynitrite, digested with trypsin, and the resulting peptides were analyzed by MALDI-TOF/TOF mass spectrometry. After the LC-MS/MS analysis, a list of scanned and identified peptides was obtained (Table S1), among which only one was identified to contain a nitrated tyrosine. Figure 5 shows the comparison of the nitrated (top) and unmodified (bottom) MS/MS spectra of these identified peptides from NADP-ME2. Nitrated peptide DAHYLTGLLPPVILSQDVQER (Z = 3) had 21 amino acids and a mass of 2409 Da (2364 Da, plus 45 Da), which is compatible with Tyr-73 nitration (Figure 5).

### 3.5. Modeling the NADP-ME2 from Arabidopsis Thaliana

The tertiary structure of NADP-ME2 from *A. thaliana* was modeled as described in the Materials and Methods section. The quality evaluation revealed that the models computed by servers RaptorX (95% coverage) and IntFold (100% coverage) gave scores, which led us to use IntFold for the final model and to carry out subsequent studies. This model was computed using Protein Data Bank (PDB) entries 2aw5 (human malic enzyme) and 1O0S (*Ascaris suum* malic enzyme complexed with NADH) as a template. These entries share 48% and 46% identity, respectively, with the sequence from *A. thaliana*; the RMSD (root-mean-square deviation) of the model with the templates being 0.62 Å with 2aw5 and 1.73 Å with 1o0s. The coordinates yielded a Qmean value [65] of 0.784, with a Qmean-Z score [66] of 0.2, and an Errat overall quality factor [63] of 78.26, and 89.3% of the residues had an averaged 3D-1D score above 0.2 (Verify3D) [62], while 93.7% of the residues were in the most favored regions in the Ramachandran plot and seven lay in outlier regions.

**Figure 6:**
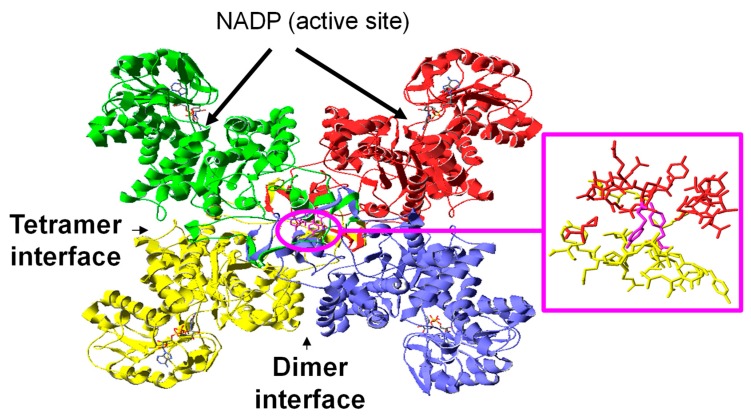
Schematic drawing of the model of the quaternary structure of the malic enzyme from *A. thaliana* showing Y73 (magenta) and details of the residues within 10 Å from Y73, which reveal its interfacial location. The four monomers are depicted in different colors.

As NADP-malic enzymes are complex molecules with a double dimer structure that yields a dimer interface and a tetramer interface, the coordinates of the biological assembly of the enzyme from *Ascaris suum* (PDB code 1O0S) were used as a template to identify the interfaces in the *A. thaliana* model (Figure 6). At this point, it is noteworthy that no further refinements were carried out and that the overlapping regions were limited to face monomers from different dimers that did not share any interface.

### 3.6. Location of the Nitrated Tyrosine Site in NADP-ME2

The malic enzyme is a dimer of the dimer with close contacts at the dimer interface, whereas the association of the two dimers to yield the tetramer is weaker. In general, the tertiary structure of all malic enzymes is similar. However, small differences may influence catalytic and regulatory mechanisms. Each monomer is divided into four domains (A, B, C, and D) that behave as rigid bodies during the conformational transition from the open to the closed form upon the binding of the divalent cation and the substrate [75].

The evolutionary analysis of the 25 Tyr residues of the NADP-ME2 sequence from *A. thaliana* shows that 15 Tyr residues are preserved in plants, with Tyr-136 being absolutely preserved through evolution in plants, mammals, and bacteria (Table S2). Through these 15 preserved residues, Tyr 158, Tyr242, Tyr457, Tyr 491, and Tyr 592 are predicted as being highly susceptible to phosphorylation (score > 0.75). Among the Tyr residues preserved in plants and predicted as non-phosphorylated, Tyr73 was identified as being nitrated by mass spectrometry. Tyr73 was a particular residue showing the highest estimated pKa (Table S2) which, according to the model, was located on the surface by protruding into the opposite monomer (Figure 6).

## 4. Discussion

Low temperature (LT) is an important environmental factor that significantly affects plant development and ROS metabolism [76]. *Arabidopsis thaliana*, as a model plant, has been used to analyze the effects of LT on physiology, gene expression, and metabolism at different levels, including photosynthesis, polyamines, and ROS, carbon, and nitrogen metabolisms [77,78,79,80,81,82,83]. Previous data have shown that NADPH regeneration by a family of NADP-dehydrogenases can prove essential as a cofactor in cellular redox homeostasis as it is an indispensable electron donor in several enzymatic reactions, biosynthetic pathways, and detoxification processes [30]. The involvement of NADPH in the metabolism of ROS and RNS has also been established as being necessary for not only the functioning of the ascorbate–glutathione cycle, via its participation in the regeneration of reduced glutathione (GSH) by glutathione reductase [84] but also for the activity of the NADPH-dependent thioredoxin system [85]. Consequently, NADPH appears to be important in the functioning of these central cell antioxidants by playing a key role in plant response to oxidative damage. Similarly, NADPH is also necessary for O_2_^•−^ generation by NADPH oxidase (NOX) [86] and is a cofactor required for NO generation by l-arginine-dependent nitric oxide synthase activity [87,88]. In addition, NADPH-generating enzymes appear to be involved in the plant response to different abiotic stresses [17,18,19,20,21,22,23])

In this study, by using *Arabidopsis thaliana* as a model, we analyzed the mechanism of response to short-term LT, specifically in relation to the status of some ROS, RNS, and NADP-malic enzyme activity. Additionally, the potential regulatory effect of tyrosine nitration on NADP-ME2 activity was analyzed in particular by an in vitro approach.

### 4.1. Differential Behaviors of NADP-Dehydrogenases, ROS, and RNS Molecules during Short-Term LT

The ROS metabolism under LT stress has been analyzed in several studies using different plant species [76,89,90,91,92,93,94,95]. More recently, occurrence of NO in higher plants and its importance under different stress conditions have become a new area of intense research to understand the role of NO and its related molecules (RNS) in the signaling events leading to plant response to LT stress conditions and their interaction with the ROS metabolism [1,11,96,97,98,99,100].

Cold acclimation in LT situations is the result of different processes, such as cryoprotectant and phospholipid production, protein stabilization, ion homeostasis maintenance, and stress situations, which are usually related to the scavenging of reactive oxygen species (ROS) [11,83]. In fact, LT-induced oxidative stress in *Arabidopsis* has been previously described and is characterized by an increase in lipid oxidation and H_2_O_2_ content, and by variation in antioxidant enzymes and the reduced functionality of catalase activity, ascorbate peroxidase (APX) and also soluble antioxidants, such as ascorbate [101,102,103]. The combined effect of less antioxidant capacity and increased ROS is associated with the damage and degradation of photosystems I and II, which finally affect plant growth [81,104]. Our finding, which indicates increased superoxide radical, falls in line closely with all these previously reported data, while our experimental LT conditions (4 °C for 48 h) led to a highly significant reduction in growth (Figure 1) accompanied by oxidative stress.

Similarly, previous data have shown that LT also provokes increased NO content in different plant organs [98,105], particularly in *Arabidopsis thaliana* leaves [96,106]. The increase in NO content observed in the roots of *Arabidopsis* seedlings also falls closely in line with these studies. However, an increase in peroxynitrite (ONOO^−^) content under LT stress conditions has not yet been reported. This increase would be expected to occur as ONOO^−^ is quickly produced by a chemical reaction that takes place between superoxide and nitric oxide radicals [107], both of which increase at LT. ONOO^−^ is the most powerful oxidant that can be produced in nitro-oxidative stress situations, such as salinity and cadmium stress [73,108], which can mediate the tyrosine nitration of proteins and affect the function of ferredoxin-NADP reductase, carbonic anhydrase, NADH-dependent hydroxypyruvate reductase, and different components of the ascorbate–glutathione cycle [37,41,47,48,109,110]. This NO-related posttranslational modification must, therefore, be a mechanism to extend the regulatory and signaling effects of NO under physiological and stress conditions.

Different NADP-dehydrogenases have been shown to be modulated by diverse types of abiotic stresses; e.g., heavy metal [17,111,112], ozone [113], salinity [18,23,114,115], drought [116], and wounding [117]. In pea plants left under natural senescence conditions in which ROS and RNS increased, we also recently identified Tyr392 to be a target of tyrosine nitration that inhibits cytosolic NADP-ICDH [42].

Under our experimental conditions, the analyzed NADP-dehydrogenase activities were differentially affected by LT in *Arabidopsis* seedlings due to the reduced activity caused by NADP-ME activity, while G6PDH and NADP-ICDH activities remained unaffected. These results suggest that NADP-ME could be the NADP-dehydrogenase enzyme that is more sensitive to the oxidation state generated by ROS and RNS production at LT stress. Interestingly, NADP-ME is also the most sensitive to H_2_S treatment in sweet pepper fruits [118]. This suggests that NADP-ME could be affected at low oxidation levels during the course of this abiotic stress, whereas the function of other dehydrogenases (G6PDH and ICDH) could remain in an attempt to maintain the cellular redox state by producing NADPH under nitro-oxidative stress. This could be crucial for plant survival under environmental insults as NADPH acts as a vital electron donor in several reductive and detoxification processes, and in antioxidant processes. Furthermore, NADPH is required in the metabolism of reactive oxygen and nitrogen species (ROS and RNS) [51] Similar to NADP-dehydrogenase enzymes, NO-PTMs differentially regulate Asc-GSH cycle components during plant response to the nitro-oxidative stress generated by salinity [47,48]. In this case, APX and monodehydroascorbate reductae (MDAR) are inactivated by tyrosine nitration, whereas glutathione reductase (GR) is not affected by this NO-PTM, which suggests a key role for GR to maintain the GSH level needed for the functioning of this antioxidant system. As the Asc-GSH cycle requires NADPH for functioning, this key antioxidant system could be regulated by tyrosine nitration at both levels: directly by regulating the function of its components; indirectly by regulating the enzymes that produce reduced power in the form of NADPH. However, these data differ from those on the behavior of NADP-dehydrogenase activities in other species exposed to LT. For example, pepper plants subjected to LT conditions (8 °C) showed an increase in the oxidative markers, such as lipid oxidation and protein nitration, together with an overall induction of the activity of major NADPH-generating systems (6PGDH, NADP-ICDH, G6PDH, and NADP-ME) after 24-hour treatments. These different results could be a result of the different exposure times to LT stress and, therefore, suggest that the oxidation level reached during this stress could be responsible for the specific modulation of NADPH-generating enzymes.

### 4.2. NADP-ME Activity Is Dysregulated by Nitration

As stated above, diverse types of environmental conditions have the capacity to modulate different NADP-dehydrogenases. Furthermore, nitro-oxidative stress situations can generate peroxynitrite (ONOO^−^) by a quick chemical reaction between superoxide radical and nitric oxide [107], which can mediate tyrosine nitration and affect the function of several proteins [47,48,109,110]. As NADPH regeneration can be a key cofactor in cellular redox homeostasis, the regulation of NADPH-generating enzymes by NO could play a key role in plant response to these adverse situations as these systems may act as a second line of defense to maintain the functioning of the main antioxidant systems [51]. Indeed, the activity of different NADPH-generating enzymes is regulated by RNS. For instance, ferredoxin-NADP reductase activity is inhibited by tyrosine nitration under high-temperature stress [41], and NADP-dependent isocitrate dehydrogenase activity is also inhibited by Tyr392 nitration, accompanied by increased ROS and RNS under natural senescence conditions [42]. When taking all these data together, tyrosine nitration seems to inhibit NADPH-generating enzymes in these situations. With NADP-EM2, Tyr73 nitration also inhibits the activity of the recombinant protein in a concentration-dependent manner. The inactivation of different NADP-generating enzymes, such as FNR, NADP-ICDH, or NADP-ME2, suggests that this NO-PTM may impair NADPH levels and, therefore, jeopardize the cellular redox state. To determine the functional role of this Tyr73 as a target of tyrosine nitration, we performed an in silico analysis. Previous reports indicate that chemical tetranitromethane (TNM) is able to mediate both cysteine oxidation and tyrosine nitration [119]. In line with this, there are also reports that TNM induces the complete inhibition of NADP-ME activity [120]. Loss of enzyme activity has been observed at pH 8.0, but not at pH 6.3, while NADP-ME has almost been 90% deactivated by incubation with an 80-fold molar excess of TNM for 5 min at 30 °C. Substrate l-malate or Mg^2+^ alone offers no protection, while NADP provided considerable protection. As reported for other NADP-dehydrogenases, in the presence of l-malate and Mg^2+^, NADP totally protects enzyme activity. This suggests that tyrosine residue may be located at or near the active site of maize NADP-ME. Spectral analysis of the modified enzyme has indicated that the modification of at least one tyrosine residue per subunit results in the complete loss of enzyme activity. The fluorescence study of unmodified and modified enzymes has postulated that the essential tyrosine residue of maize NADP-ME is possibly involved in l-malate binding. By using knockout *Arabidopsis* mutants of NADP-ME2, it has been recently suggested that this enzyme does not play a key role in the response mechanism to oxidative stress [121]. In this context, our data indicated that this enzyme was negatively modulated by several RNS produced under nitro-oxidative stress conditions caused by short-term LT. This could partly explain why NADP-ME does not appear to be involved in oxidative stress because it appears to be partially deactivated [121].

Tyrosine nitration is a NO-mediated posttranslational modification (NO-PTM) that, in some cases, modulates the function of a specified antioxidant enzyme [37,122]. For example, for pea cytosolic APX, a key enzyme for regulating H_2_O_2_, cellular levels under salinity and nitro-oxidative-associated stress conditions, the enzyme is jointly regulated by this NO-PTM. Thus peroxynitrite inhibits APX activity by the nitration of Tyr235, which is located at the bottom of the pocket where the haem group is enclosed [48]. Furthermore, MDAR activity, another component of the Asc-GSH cycle, is also inhibited by Try345 nitration, which could be involved in the cofactor binding site [47]. Interestingly, NADP-ICDH activity is also down-regulated by Tyr392 nitration as a result of the oxidative process generated during the natural senescence of pea plant roots. In this case, the nitration process also appears to be involved in the disruption of the enzyme-cofactor interaction [42]. In addition, peroxisomal hydroxypyruvate reductase, an enzyme involved in the photorespiration process, is down-regulated by tyrosine nitration. In this case, Tyr 198 nitration has been proposed to be responsible for this inhibition [110]. In this context, NADP-ME regulation by tyrosine nitration is another example of the key role played by tyrosine nitration in the regulation of the target protein function. It is noteworthy that this process becomes relevant when it regulates crucial processes in plants, such as antioxidant defense systems. However, the regulation mechanism is not always straightforward and, for NADP-ME, modulation is especially complex [75].

Tyr73 is a particular residue as determined by both location and estimated pKa. Residue Tyr73 is at a contact distance from Glu11 and Asp12 from the same monomer, and Glu78, Asp70, Tyr 587, and Arg588 from the neighbor monomer. The mechanism underlying the effect of Tyr73 nitration on functionality may be the disruption of these interactions as it has been reported for the pigeon malic enzyme that the residues located at the N-end play an important role in subunit interactions and Mn(II) malate binding [123].

## 5. Conclusions

The present data show that short-term LT stress affects the metabolism of both ROS and RNS in *Arabidopsis* seedlings, which appears to be accompanied by nitro-oxidative stress. Of all the different analyzed NADP-dehydrogenases, cytosolic NADP-ME was negatively modulated under LT conditions. In this context, the in vitro analysis that used recombinant *Arabidopsis* cytosolic NADP-ME2 treated with peroxynitrite enables us to demonstrate that this enzyme is negatively modulated by nitration, which falls in line with the reduction observed in NADP-ME activity under short-term LT stress and also limits NADPH supply. At the molecular level, Tyr73 was identified as the most likely residue to be involved in this negative NADP-ME regulation by this NO-PTM.

## Supplementary Materials

The following are available online at https://www.mdpi.com/2076-3921/8/10/448/s1, Table S1: List of peptides scanned and peptides identified by LC-MS/MS in the recombinant NADP-ME2 from *Arabidopsis thaliana*, Table S2: Evolutionary conservation of the Tyr residues in ME related sequences found in UniprotKB data base.
