# Supplementary material for: Short-Term Low Temperature Induces Nitro-Oxidative Stress that Deregulates the NADP-Malic Enzyme Function by Tyrosine Nitration in Arabidopsis thaliana
Source: Antioxidants (Basel). 2019 Oct 1;8(10):448. doi: 10.3390/antiox8100448 (PMC6827146; doi:10.3390/antiox8100448)
Supplement: Supplementary file 1 [file antioxidants-08-00448-s001.zip › Supplemental Table 2.pdf]

Supplementary Table S2. Evolutionary conservation of the Tyr residues in ME related sequences found in UniprotKB data base (Release 2014_10) subsections Viridiplantae, Mammalia and Archaea and prediction of phosphorylation and pKa of the ME from A. thaliana. For each Tyr residue, both rho score and the sequence variants are compiled. Experimentally detected nitrated Tyrosine residue is shown underlined and in bold. Rho values deviate from 1 as variability increases and NetPhos score tends to 1 as the likelihood of phosphorylation is maximum.

Residues conservation

Residue	Viridiplantae	Mammalia	Archaea	NetPhos Score	pKa
Y28	YNQ 2.09	R.LQSAVFIY 12.02	.RSY 9.27	0.203	15.0
Y50	YSCNHF 7.76	Y.KHIASFCQ 14.39	.IARY 9.27	0.240	8.9
Y58	YHLF 6.40	L.SEIPFTY 11.81	DK.LY 4.44	0.662	11.4
**<u>Y73</u>**	Y 1.00	GNKY 3.35	.GSEY 9.27	0.294	17.5
Y99	Y 1.00	QLMIY 5.14	AKTY.NQE 7.60	0.034	11.0
Y106	Y 1.00	Y 1.00	AS.NY 3.86	0.026	10.5
Y121	Y 1.00	Y 1.00	VTGMLIAFY 7.42	0.021	14.3
Y136	Y 1.00	Y 1.00	Y 1.00	0.010	19.9
Y147	Y 1.00	Y 1.00	IDYH 3.23	0.437	13.3
Y158	YF 4.55	FY 1.55	YF 2.86	0.774	14.0
Y211	Y 1.00	Y 1.00	FY 1.58	0.219	15.5
Y242	Y 1.00	Y 1.00	.Y 9.27	0.795	15.5
Y255	Y 1.00	Y 1.00	.Y 9.27	0.403	15.8
Y270	Y 1.00	FY 2.17	FY 1.53	0.120	12.8
Y293	Y 1.00	Y 1.00	LAY 2.43	0.202	13.9
Y457	YH 1.97	Y 1.00	KELIY 4.12	0.848	13.4
Y477	YF 2.82	LY 1.06	.IVY 9.27	0.080	10.0
Y482	YFLH 9.42	FLY 2.52	DIY 2.48	0.048	10.9
Y491	Y 1.00	Y 1.00	MLIFY 5.02	0.805	13.7
Y529	YFL 6.82	LIY 1.56	.PDY 9.27	0.984	10.0
Y535	YF 1.92	Y 1.00	IVLY 3.07	0.032	14.4
Y557	Y 1.00	YF. 2.34	CIQVY 5.97	0.027	11.1
Y580	Y 1.00	YFW. 3.41	KGSLAEWY 6.47	0.556	13.2
Y584	Y 1.00	Y. 3.28	SKNREY 5.81	0.563	12.5
Y587	YF 3.94	F.IVLMY 6.89	RKSLYI 5.73	0.006	15.2
