# Supplementary material for: Short-Term Low Temperature Induces Nitro-Oxidative Stress that Deregulates the NADP-Malic Enzyme Function by Tyrosine Nitration in Arabidopsis thaliana
Source: Antioxidants (Basel). 2019 Oct 1;8(10):448. doi: 10.3390/antiox8100448 (PMC6827146; doi:10.3390/antiox8100448)
Supplement: Supplementary file 1 [file antioxidants-08-00448-s001.zip › Supplemental Table 1.pdf]

Supplementary Table S1. List of peptides scanned and peptides identified by LC-MS/MS in the recombinant NADP-ME2 from Arabidopsis thaliana.

Peptides identified[(1)]	Peptides scanned	Length (AA)	#Tyr
AIFGSGSPFDPVVYDGK	AIFGSGSPFDPVVYDGK	17	1
DAHYLTGLLPPVILSQDVQER	DAHYLTGLLPPVILSQDVQER	21	1
	DDMLLAASEALAAQVTEE HYANGLIYPPFSNIR	33	2
EISANIAACVAAK			
FAESSMYSPVYR	FAESSMYSPVYR	12	2
ILGLGDLGCQGMGIPVGK			
KPQGLYISLNEK	KPQGLYISLNEK	12	1
LLIDNVEELLPVVYTPTVGEACQK	LLIDNVEELLPVVYTPTVGEACQK	24	1
LLNDEFYIGLK	LLNDEFYIGLK	11	1
	LSLYTALGGIRPSACLPITIDVGTNN EK	28	1
	QYTVPLQR	8	1
RATGQEYAEFLHEFMCAVK	RATGQEYAEFLHEFMCAVK	19	1
SGVGGGISDVYGEDSATLD QLVTPWVTSVASGYTLMR	SGVGGGISDVYGEDSATLD QLVTPWVTSVASGYTLMR	37	2
TYDLGLASNLPR	TYDLGLASNLPR	12	1
TYLPGQANNCYIFPGLGLG LIMSGAIR	TYLPGQANNCYIFPGLGLG LIMSGAIR	27	2
	YMALMDLQER	10	1
YSDSHLVFNDDIQGTASVV LAGLIAAQK	YSDSHLVFNDDIQGTASVV LAGLIAAQK	28	1

(1) Some of the detected peptides do not contain tyrosines. These peptides were not included in the targeted MS/MS detection. They were detected and identified because their molecular weight coincides with that of expected peptides.
